# Mathematical Model to Predict Skin Concentration after Topical Application of Drugs

**DOI:** 10.3390/pharmaceutics5040634

**Published:** 2013-12-16

**Authors:** Hiroaki Todo, Takeshi Oshizaka, Wesam R. Kadhum, Kenji Sugibayashi

**Affiliations:** Faculty of Pharmaceutical Sciences, Josai University, 1-1 Keyakidai, Sakado, Saitama 350-0295, Japan; E-Mails: ht-todo@josai.ac.jp (H.T.); t-oshizaka@nichiban.co.jp (T.O.); rkwesam@josai.ac.jp (W.R.K.)

**Keywords:** skin permeation, skin concentration, silicone membrane, permeation profile

## Abstract

Skin permeation experiments have been broadly done since 1970s to 1980s as an evaluation method for transdermal drug delivery systems. In topically applied drug and cosmetic formulations, skin concentration of chemical compounds is more important than their skin permeations, because primary target site of the chemical compounds is skin surface or skin tissues. Furthermore, the direct pharmacological reaction of a metabolically stable drug that binds with specific receptors of known expression levels in an organ can be determined by Hill’s equation. Nevertheless, little investigation was carried out on the test method of skin concentration after topically application of chemical compounds. Recently we investigated an estimating method of skin concentration of the chemical compounds from their skin permeation profiles. In the study, we took care of “3Rs” issues for animal experiments. We have proposed an equation which was capable to estimate animal skin concentration from permeation profile through the artificial membrane (silicone membrane) and animal skin. This new approach may allow the skin concentration of a drug to be predicted using Fick’s second law of diffusion. The silicone membrane was found to be useful as an alternative membrane to animal skin for predicting skin concentration of chemical compounds, because an extremely excellent extrapolation to animal skin concentration was attained by calculation using the silicone membrane permeation data. In this chapter, we aimed to establish an accurate and convenient method for predicting the concentration profiles of drugs in the skin based on the skin permeation parameters of topically active drugs derived from steady-state skin permeation experiments.

## 1. Introduction

Skin has been the focus as an application site of cosmetics and therapeutic drugs. Many transdermal drug delivery systems and topical drug formulations as well as cosmetics are on the market and determining the percutaneous absorption of drugs and cosmetic ingredients is important for develop good topical formulations; however, percutaneous absorption or skin permeation itself is not always important for topical formulations [1]. The concentration-dependent pharmacological activity of a metabolically stable drug that binds with specific receptors of known expression levels in an organ can be determined by Hill’s equation [2]. However, predicting the biological effects, either therapeutic or toxic, of a drug whose bio-disposition is complicated by metabolic events is based on its concentration at its site of action. The efficacy of topical drug delivery to the peripheral blood circulation is based on the concentration of the drug in the blood [3,4,5]. On the other hand, the blood concentration-time course can be predicted using skin permeation parameters, such as flux and permeability coefficients, obtained from *in vitro* skin permeation experiments [6,7,8].

Several techniques have been developed to measure the sectional skin concentrations of drugs, including suction blisters [9], punch and shave biopsies [10], heating [11], autoradiography [12], and tape-stripping [13,14,15]. However, measuring drug concentrations in small well-defined segments of the skin is very challenging.

A few studies have determined drug levels in small segments of the skin using radiolabeled drugs. To our knowledge, no attempts have been made to predict drug concentrations in depth-dependent sections of the skin, except for one [16] in which already published results were fitted to selected models.

Recently, criticism against animal experiments has greatly increased from the viewpoint of animal welfare. In the EU, animal experiments to the production and import of cosmetic formulations are banned from 2009 to 2013 [17,18,19,20]. Scientists have to be aware of the spirit of the 3Rs for animal experiments, where the 3Rs are “Reduction”, referring to methods that enable researchers to obtain comparable levels of information from fewer animals, or to obtain more information from the same number of animals, “Refinement” refers to methods that alleviate or minimize potential pain, suffering or distress, and enhance the welfare of the animals used, and “Replacement” refers to the preferred use of non-animal methods over animal methods whenever it is possible to achieve the same scientific aims.

We therefore used silicone membrane as an alternative to skin membrane to determine the skin concentration of cosmetic ingredients or drugs, from the viewpoints of the great necessity of estimating their concentration in the skin in the development of cosmetics or topical formulations as well as animal experiment alternatives. Silicone membrane has been used as an alternative to skin membrane and the permeation of cosmetic ingredients and drugs through the silicone membranes was compared with that through animal skin [21,22,23,24]. Few experiments have been performed, however, on their skin or membrane concentration. In the present chapter, a method for estimating skin concentration was established using silicone membrane permeability. A series of parabens (methyl, ethyl, *n*-propyl, *n*-butyl esters) were used as model penetrants, since they have very different lipophilicities (*i.e.*, *n*-octanol/water partition coefficient) in spite of having a similar molecular weight (152–194 Da). 

## 2. Theoretical

### 2.1. One-Layered Diffusion Model

#### 2.1.1. Determination of Membrane Concentration

**Figure 1:**
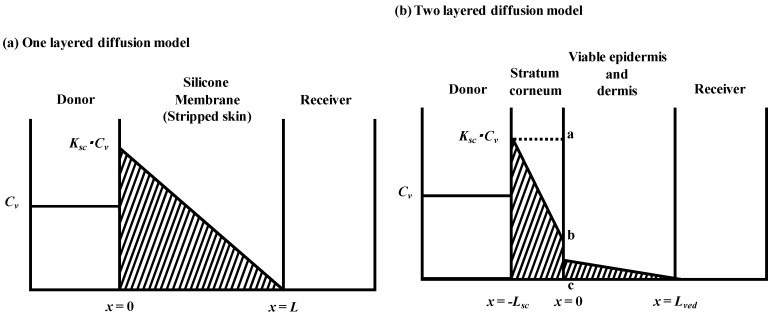
Schematic diagram of concentration-distance profile in one layered (**a**) and two layered (**b**) diffusion membrane models in membrane permeation experiments. Membrane thickness is *L* and Lsc + Lved for one- and two-layered diffusion membranes models, respectively. *C*_v_ is the donor concentration of parabens and *K* and *K*_sc_ are partition coefficients to membranes.

The diffusion of chemical compounds in the membrane is expressed theoretically by Fick’s 2nd law of diffusion, 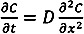, under the assumption that the membrane is a homogeneous single layer, where *C* is the penetrant concentration in the membrane at position, *x*, and time, *t* [25,26,27]. When a sink condition is assumed on the receiver side of the membrane, *i.e.*, *x* = *L*, a set of initial conditions (*C* = 0 at *t* = 0 and 0 < *x* < *L*) and boundary conditions (*C* = *KC*_v_ at *x* = 0 and *C* = 0 at *x* = *L*, where *K* is the partition coefficient of the penetrant from the vehicle to membrane and *C*_v_ is the penetrant concentration in the vehicle) are obtained (see Figure 1a).

Then, membrane concentration, *C*, and its steady-state level at an infinite time, *C*_ss_, are expressed as follows [28]:

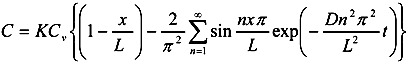
(1)

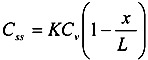
(2)

Further, the mean membrane concentration of penetrant, *C* and *C_ss_* are obtained by integrating *C* in Equations (1) and (2) from *x* = 0 to *L*:

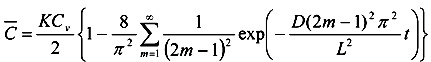
(3)

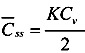
(4)

As shown in Equation (4), the mean membrane concentration of the penetrant can be determined by *K* and *C*_v_, not by *D*. Because *C*_v_ is a known parameter, a parameter, *K*, is the only determinant of the mean membrane concentration of the penetrant.

#### 2.1.2. Determination of Membrane Permeation

Since the silicone membrane can be supposed to be homogenous in one layer, the permeation profiles of parabens throughout the membrane were analyzed using a one-layered diffusion model [29,30]. Under the initial and boundary conditions shown as above, the amount of drug permeating the unit area of the silicone membrane at time *t*, *Q*, can be represented as,

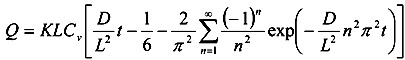
(5)

The partition parameter (*KL*) and diffusion parameter *(D*/*L*^2^) were obtained by curve fitting the obtained data to Equation (5) using the least squares method. In the calculation of *D* and *K*, the thickness of the silicone membrane was fixed at 68 μm. Permeability coefficient, *P*, was calculated by an Equation, 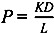 .

Stripped hairless rat skin was treated as a homogeneous layer, the same as the silicone membrane.

### 2.2. Two-Layered Diffusion Model

#### 2.2.1. Determination of Membrane Concentration Using Two-Layered Diffusion Model

Figure 1b shows a typical two-layered diffusion model of skin. Penetrant concentration in the first layer of skin, the stratum corneum, *C*_sc_, and that in the 2nd layer, the viable epidermis and dermis of skin, *C*_ved_, can be expressed by Fick’s 2nd law of diffusion. Similarly, initial conditions and boundary conditions are represented. Boundary condition between the first and second layer is *C*_ved_ = *K*_sc_*K*_ved_*C*_sc_ and 
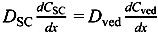
 , where *K*_sc_ and *K*_ved_ are partition coefficients of the penetrant from the vehicle to stratum corneum and to the viable epidermis and dermis, and *D*_sc_ and *D*_ved_ are diffusion coefficients in the stratum corneum and viable epidermis and dermis, respectively.

In the two-layered diffusion model, the overall permeability coefficient, *P*_tot_, can be expressed by that in the stratum corneum, *P*_sc_, and in the viable epidermis and dermis, *P*_ved_, as follows, in Equation (6) [31]:

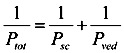
(6)

In addition, the reciprocal of the permeability coefficient can be replaced with permeation resistant, *R* and the following equation can be derived, as Equation (7).



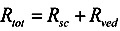
(7)

The permeation resistance of the penetrants can be represented as in the electric circuit. Two resistances, *R*_sc_ and *R*_ved_ exist in the skin membrane, as shown in Figure 1b. The ratio of the Line segment *ab* against *bc* at the surface between the stratum corneum and viable epidermis, *x* = 0, should be the ratio of *R*_sc_ against *R*_ved_. Thus, the penetrant concentration at point *b*, *C*_b_, can be represented by:

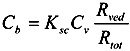
(8)

The amount of penetrant in the unit area of the stratum corneum, *M*_sc_, can be represented using Equation (8) as Equation (9).


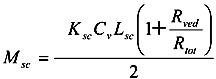
(9)

Since the partition coefficient of the penetrant from the stratum corneum to the viable epidermis and dermis is represented as *K*_sc_/*K*_ved_, the amount of penetrant in the unit area of the viable epidermis and dermis, *M*_ved_, can be represented as,

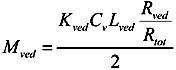
(10)

By summation of Equations (9) and (10), *M*_tot_, the drug amount in the unit area of the skin can be represented as follows:

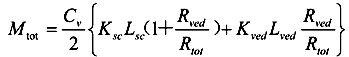
(11)

By dividing *M*_tot_ in Equation (11) by skin thickness, *L*_tot_, the average drug concentration in the skin, *C_ss_*, is

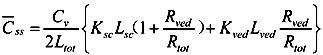
(12)

When resistances, *R* are changed to permeability coefficients, *P*, finally Equation (13) was derived,

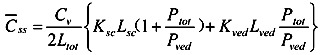
(13)

#### 2.2.2. Determination of Membrane Permeation Using Two-Layered Diffusion Model

Two diffusion coefficients, *D*_sc_ and *D*_ved_ and two partition coefficients, *Ksc* and *Kved* were obtained by curve fitting the cumulative amount of parabens that permeated through the full-thickness skin and stripped skin to the theoretical values using the least squares method, where theoretical values were expressed by two diffusion equations (Fick’s 2nd law) showing the diffusion profiles in the stratum corneum and viable epidermis and dermis. Differential equations describing Fick’s 2nd law are as follows:

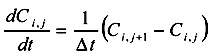
(14)

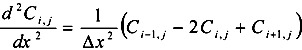
(15)

Mathematical treatment for determining the skin permeation using two-layered diffusion model was the same as in our previous method [26].

### 2.3. Calculation of Permeation Parameters

Generally, most resistance against drug permeation throughout hairless rat skin is in the stratum corneum [31]. Rat skin was also treated as a single-layered membrane, the same as the silicone membrane. Parameters *D* and *K* were calculated under the assumption that the stratum corneum is a homogeneous membrane with a thickness of 15 μm.

### 2.4. Calculation of Theoretical Membrane Concentration of Parabens

The theoretical membrane concentration of parabens was calculated by Equation (6) and the *K* value obtained from the membrane permeation study. 

## 3. Materials and Methods

### 3.1. Reagents and Materials

Parabens:methyl paraben (MP), ethyl paraben (EP), *n*-propyl paraben (PP), *n*-butyl paraben (BP), were obtained from Tokyo Kasei Chemical Co., Ltd. (Tokyo, Japan). An esterase inhibitor, diisopropyl fluorophosphate (DFP), and a deproteinization agent, trichloroacetic acid (TCA), were obtained from Wako Pure Chemical Industries, Ltd. (Osaka, Japan). Other reagents and solvents were liquid chromatograph and special grade chemicals.

The silicone membrane (Dow Corning 7-4107) was a gift from Nagase & Co., Ltd. (Tokyo, Japan).

### 3.2. Experimental Animals

Male hairless rats (WBM/ILA-Ht, 230–280 g) were obtained from the Life Science Research Center, Josai University (Sakado, Saitama, Japan) or Ishikawa Experimental Animal Laboratories (Fukaya, Saitama, Japan). All animal experiments were performed according to the ethics committee of Josai University.

### 3.3. Membrane Permeation Experiments of Parabens

**Figure 2:**
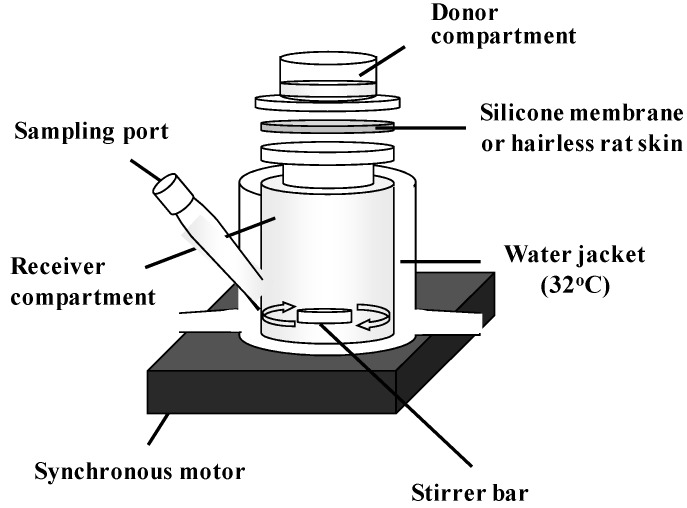
Schematic representation of permeation experiment using silicone membrane and hairless rat skin.

The silicone membrane was set on a Franz-type diffusion cell (receiver cell volume: 6.0 mL, effective diffusion area: 1.77 cm^2^) using cyanoacrylate glue [19,20,21,22]. Phosphate-buffered saline (pH 7.4, PBS) was applied to the receiver cell and maintained at 32 °C for 15 min. Aliquots (0.5 mL) of different concentrations of parabens (MP 10 mM, EP 5 mM, PP 1 mM, BP 0.5 mM) in PBS were applied to the donor cell to start the permeation experiment. The experimental setup is shown in Figure 2. The receiver solution was stirred on a magnetic stirrer with a bar, the experiment was performed at 32 °C. At predetermined intervals, 400 μL of the receiver solution was sampled, and the same volume of PBS was added to the receiver cell to keep the volume constant. Paraben concentration was determined by HPLC. 

Abdominal skin (full-thickness skin or stripped skin) was excised from hairless rats under pentobarbital (25 mg/kg, i.p.) anesthesia and debris and excess fat were trimmed off the excised skin. Stripped skin was made by tape-stripping the stratum corneum 20 times before excising from rats. The obtained skin piece was then set on the Franz-type diffusion cell, as above. DFP (2.7 μmol/mL in PBS) was applied to the receiver cell for half an hour in order to inhibit esterase enzymes activity [12,24,25,26]. After rinsing off the reagent, paraben solution (0.5 mL) in PBS and DFP in PBS (0.54 μmol/mL, 6.0 mL) were added to the donor and receiver cells, respectively, to start the skin permeation experiment. Other procedures were as in the silicone membrane permeation experiments.

### 3.4. Determination of Extraction Ratio of Parabens

#### 3.4.1. Silicone Membrane

Silicone membrane was loaded with parabens in chloroform and dried. Fresh chloroform (1 mL) was then applied to the silicone membrane piece. After agitating for 15 min, the resulting chloroform was sampled and fresh chloroform was again added for the second extraction of parabens. Total chloroform containing parabens was evaporated to dryness and the sample was reconstituted with 1.0 mL acetonitrile. The sample was injected onto HPLC to determine the paraben concentration. The extraction ratio of parabens from silicone membrane was almost 1.0. 

#### 3.4.2. Hairless Rat Skin

Hairless rat skin was loaded with parabens in water, and the skin piece was minced with scissors and homogenized at 12,000 rpm using a homogenizer (Polytron PT-MR 3000; Kinematica Inc., Littau, Switzerland) for 5 min at 4 °C. The homogenate was incubated for 1 h at 32 °C. The same volume of 16% TCA was added to the skin homogenate and agitated for 15 min. The supernatant after centrifugation (15,000 rpm, 5 min, 4 °C) was injected onto HPLC. Parabens were extracted from the skin homogenate using chloroform, as in the silicone membrane experiment. The extraction ratio of parabens was almost 1.0.

### 3.5. Determination of Paraben Concentration in Silicone Membrane and Hairless Rat Skin

#### 3.5.1. Silicone Membrane

After the permeation experiment, the donor solution was removed and the silicone membrane was washed with PBS (1 mL). Chloroform (1 mL) was added to the silicone membrane in the Franz-type diffusion cell (permeation area: 1.77 cm^2^) and agitated for 15 min to extract parabens from the membrane. The subsequent procedure was as in Section 3.4.

#### 3.5.2. Hairless Rat Skin

After the permeation experiment, the donor solution was removed and the stratum corneum side of hairless rat skin was washed two times with PBS (1 mL). The rat skin was taken from the diffusion cell, and the permeation area of the skin (1.77 cm^2^) was kept in a freezer (−15 °C) before determining the skin concentration. This frozen skin was minced with scissors and PBS (1 mL) was added to homogenize the minced skin (12,000 rpm, 5 min, 4 °C). The subsequent procedure was as in Section 3.4.

### 3.6. Determination Methods of Parabens

The same volume of acetonitrile containing an internal standard (another paraben) was added to the paraben samples. After slight mixing, the sample was injected onto HPLC. The HPLC system consists of a pump (LC-10AD; Shimadzu, Kyoto, Japan), Chromatopac (C-R6A; Shimadzu), UV detector (SPD-6A; Shimadzu), system controller (SCL-6B; Shimadzu) and an auto-injector (SIL-7A; Shimadzu). The column was LiChroCART^®^250-4 (KGaA·64271; Merck, Darmstadt, Germany) kept at 40 °C during the eluting mobile phase, 0.1% phosphoric acid:acetonitrile = 75:25 for MP and EP, 0.1% phosphoric acid:acetonitrile = 55:45 for PP and BP. The flow rate was 1.0 mL/min. The injection volume was 20 μL, and detection was performed at 260 nm.

## 4. Results and Discussions

### 4.1. Partition Coefficient and Concentration of Parabens into and in the Silicone Membrane

Since drug distribution in the skin membrane is a physical phenomenon, it can be evaluated using artificial membranes as well as animal skin. A silicone membrane was used in this experiment due to its cost and easy availability.

The direct measurement of drug concentration in the membrane has several problems. Generally, only one data point is obtained from one membrane after drug application. In addition, controlling the removal of the drug formulation from the membrane surface is very difficult. Hard cleaning of the membrane surface decreases the membrane concentration, whereas inadequate cleaning may leave the drug formulation on the membrane. We first performed the membrane permeation experiment and permeation parameters were obtained. The membrane concentration can be calculated using the partition coefficient, *K*, of the applied drug from the vehicle to the membrane, as shown in Equation (6). Next, the calculated values were compared with the directly observed membrane concentration. The membrane was obtained after the membrane permeation experiments.

**Figure 3:**
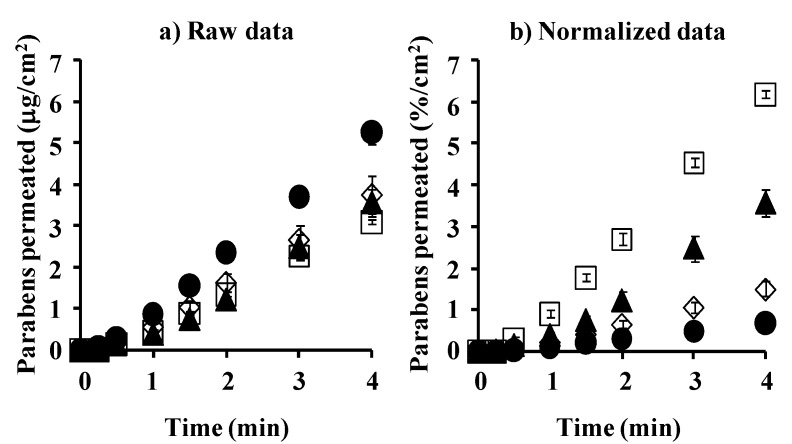
Time course of the cumulative amount of parabens that permeated the silicone membrane. (**a**) raw data; (**b**) normalized data. ●: 10 mM MP; ◊: 3 mM EP; ▲: 1 mM PP; □: 0.5 mM BP. Each data point represents the mean ± S.E. (*n* = 4–8).

Figure 3 shows the cumulative amount of parabens that permeated the silicone membrane. Figure 3a,b show raw and normalized data (raw data divided by application concentration of parabens), respectively. The permeation ratio of BP against the application amount was highest, followed by PP, EP and MP. The increase in the lipophilicity of parabens increased the permeability, as shown in Figure 3b.

Generally, high permeability and solubility of chemicals through and in the membrane are observed when the solubility parameter of chemicals is close to that of membrane. The solubility parameter values of parabens were calculated by Feder’s equation. The solubility parameter of BP, 10.9 (cal/cm^3^)^1/2^, is closest to that of the silicone membrane (7.3–7.5 (cal/cm^3^)^1/2^) among the parabens (MP: 11.5 (cal/cm^3^)^1/2^, EP: 11.3 (cal/cm^3^)^1/2^, PP: 11.1 (cal/cm^3^)^1/2^) used in this experiment.

**Figure 4:**
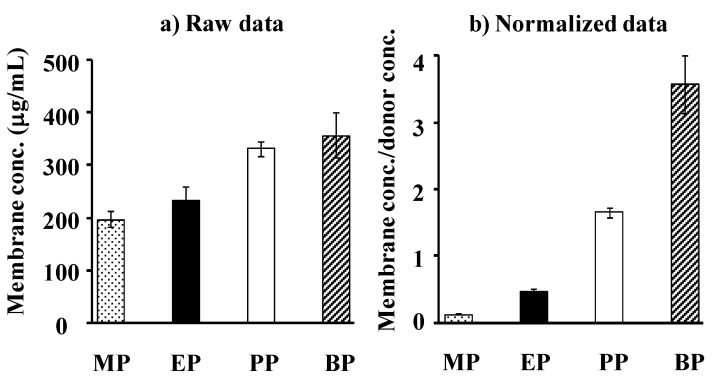
Raw data (**a**) and normalized date [(silicone membrane concentration)/(donor concentration)] (**b**) for steady-state silicone membrane concentration of parabens. Each column represents the mean ± S.E. (*n* = 4–8).

Figure 4a,b show the observed concentration and normalized concentration corrected using the application concentration of parabens in the silicone membrane, respectively. Each concentration is that at the steady state after starting the permeation experiments. An increase in *K*_o/w_ of parabens increases the membrane concentration.

**Figure 5:**
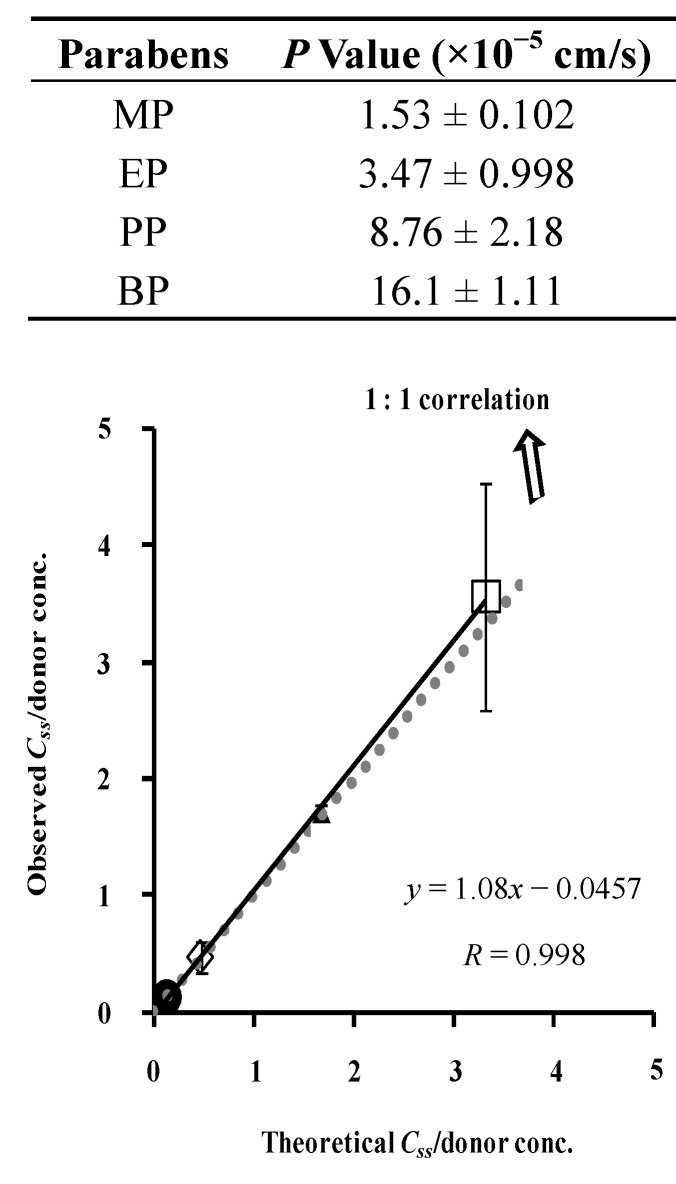
Relationship between theoretical and observed steady-state silicone membrane concentration of parabens. Normalized data were used (see Figure 4). One-layered diffusion membrane model was used to obtain theoretical steady-state membrane concentration of parabens. ●: 10 mM MP; ◊: 3 mM EP; ▲: 1 mM PP; □: 0.5 mM BP. The observed data represent the mean ± S.D. (*n* = 4–8). Almost 1:1 correlation was found. *P* values were listed below.

Figure 5 shows the relationship between the theoretical and observed values of paraben concentration in the silicone membrane. This figure shows almost a 1:1 relationship between them, suggesting that the silicone membrane can be assumed to be one homogenous layer and that the membrane concentration of parabens can be theoretically determined by *C*_v_ and *K*. These results also suggest that the permeation experiment is useful to determine the membrane concentration. The figure also contains the permeation parameters of parabens in the legend. The permeation parameters were obtained by curve-fitting the permeation profiles to Fick’s law of diffusion using the nonlinear least squares method, under the assumption that the silicone membrane is one homogenous layer. The permeability coefficient, *P*, of MP and BP was lowest and highest among the parabens used in this experiment. The diffusion coefficient, *D*, of these parabens in the silicone membranes was not so different (only 2–3 times different), whereas partition coefficient, *K*, was very different among these parabens (26 times different between MP and BP). A very different permeability coefficient, *P*, is closely related to the *K* of parabens. Since the *p*-value of parabens throughout the silicone membrane was very high (about 10^−5^ cm/s), the permeation profiles can be evaluated in a short experimental period. The theoretical membrane concentration of parabens, calculated from the *K*-value was very close to the observed membrane concentration.

### 4.2. Membrane Permeation and Concentration: Comparison between Silicone Membrane and Animal Skin

The theoretical concentration of parabens in silicone membrane, which was calculated from partition coefficient, *K*, was close to the observed concentration. A similar trial was carried out for the paraben concentration in hairless rat skin. Partition and skin concentration of parabens in rat skin were compared to those in the silicone membrane.

**Figure 6:**
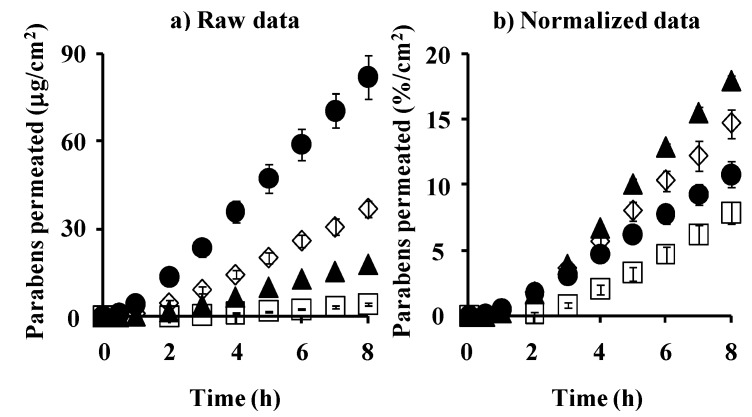
Time course of the cumulative amount of parabens that permeated hairless rat intact skin. (**a**) raw data; (**b**) normalized data. Symbols: ●: 10 mM MP; ◊: 3 mM EP; ▲: 1 mM PP; □: 0.5 mM BP. Each data point represents the mean ± S.E. (*n* = 5–11).

Figure 6 shows the cumulative amount of parabens that permeated the excised hairless rat skin. Figure 6a,b show the mean raw data and normalized permeation data. The latter was normalized by the application amount of parabens. The raw skin permeation data of parabens, as shown in Figure 6a, are similar to silicone membrane permeation; however, the permeability coefficient ratio of BP against MP in hairless rat skin was much larger than that in the silicone membrane. This may have been due to the different permeation pathways between the silicone membrane and rat skin. The silicone membrane is a homogeneous membrane, whereas hairless rat skin has appendages such as hair follicles as well as the stratum corneum pathway. Interestingly, BP permeation through the silicone membrane was highest in the normalized data (Figure 3b) among the parabens used in the present study, whereas PP permeation through rat skin was highest (Figure 6b). These data are probably due to similar solubility parameter of BP or PP to that of the silicone membrane or rat skin, respectively.

**Figure 7:**
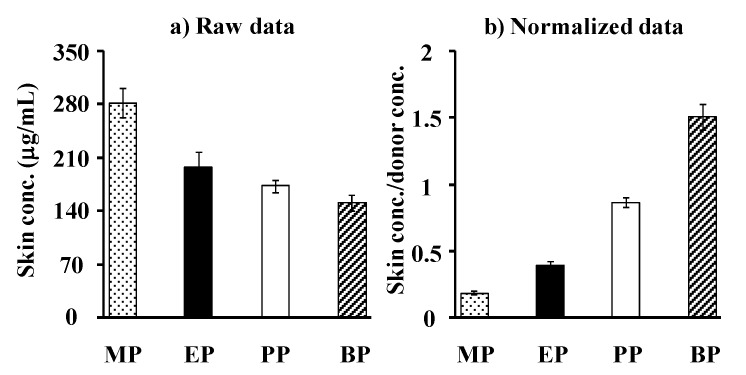
Raw data (**a**) and normalized data (**b**) [(skin concentration)/(donor concentration)] for steady-state hairless rat skin concentration of parabens. Each column represents the mean ± S.E. (*n* = 5–11).

Figure 7a,b show the observed and normalized concentrations of parabens corrected using their application concentrations in hairless rat skin, respectively. Each paraben concentration in rat skin was that at the steady state after starting the skin permeation experiment. As in the silicone membrane, the increase in partition coefficient, *K*, of parabens increased the skin concentration. The increment, however, was marked in the silicone membrane concentration, not in rat skin, except MP. This is due to the similar solubility parameter of parabens to that in the silicone membrane.

**Figure 8:**
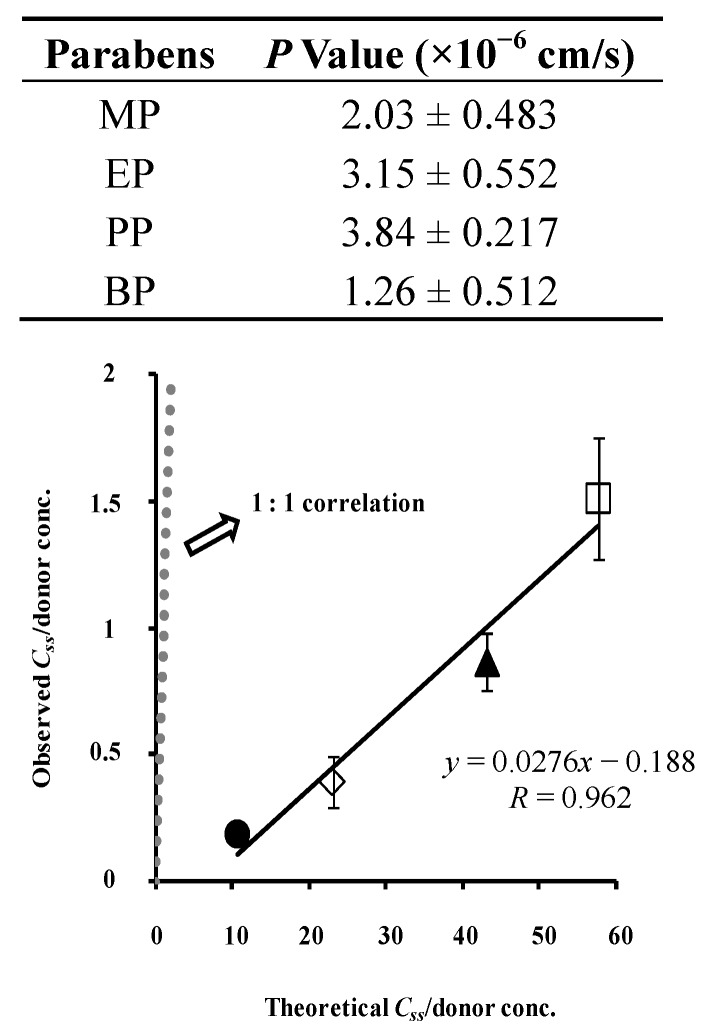
Relationship between theoretical and observed steady-state hairless rat skin concentration of parabens. Normalized data were used (see Figure 7). One-layered diffusion membrane model was used to obtain theoretical steady-state membrane concentration of parabens. ●: 10 mM MP; ◊: 3 mM EP; ▲: 1 mM PP; □: 0.5 mM BP. The observed data represent the mean ± S.D. (*n* = 5–11). The obtained line was very different from 1:1 correlation. *P* values were listed below.

Figure 8 shows the relation between the theoretical and observed skin concentrations of parabens. Although no 1:1 relationship was observed, a linear relation was found. When skin permeation data of a series of compounds are obtained, the skin concentration of the compounds may be theoretically calculated. The figure also contains the obtained permeation parameters of parabens in the figure legend, under the assumption that the skin membrane is one homogenous layer. Compared to the silicone membrane, lower *P* and *D* and higher *K* were observed in the rat skin. Unfortunately, the theoretical skin concentration of parabens was much higher than the observed skin concentration. This lack of a 1:1 relationship is due to the assumption that the skin is one homogenous layer.

**Figure 9:**
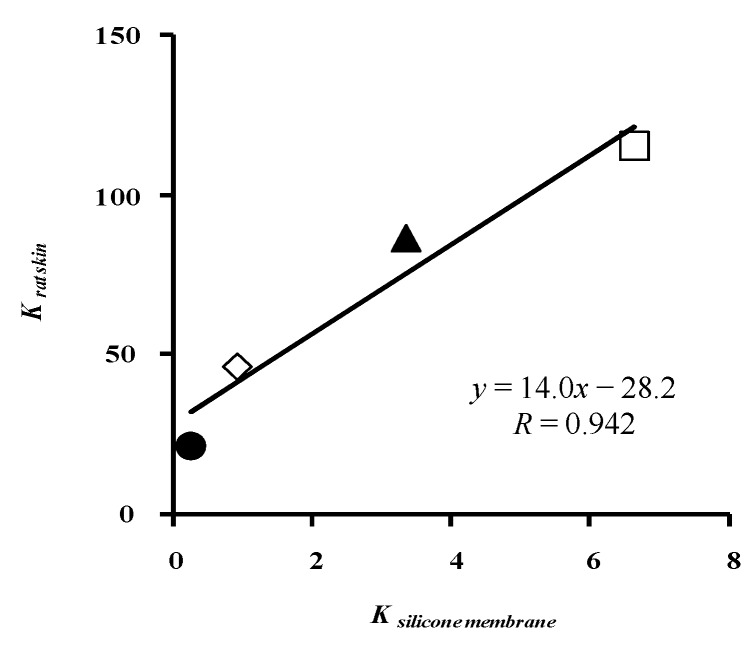
Relationship between *K* to silicone membrane and *K* to rat skin (one-layered diffusion model). ●: 10 mM MP; ◊: 3 mM EP; ▲: 1 mM PP; □: 0.5 mM BP. Each data point represents the mean ± S.E. (*n* = 4–8 in silicone membrane data and 5–11 in rat skin data).

Figure 9 illustrates the relationship between *K* and silicone membrane and rat skin. The two *K* values have a linear relation. These results suggest that the skin concentration of parabens cannot be easily predicted by calculating the skin permeation profiles because of the simple assumption about the skin membrane.

### 4.3. Simulation of Skin Concentration of Parabens

We then assumed that skin consists of two diffusion layers, of which the first layer is the stratum corneum and the second layer is the viable epidermis and dermis. The partition coefficient from the vehicle to the stratum corneum and that to the viable epidermis and dermis was obtained from permeation data through full-thickness and stripped skin.

**Figure 10:**
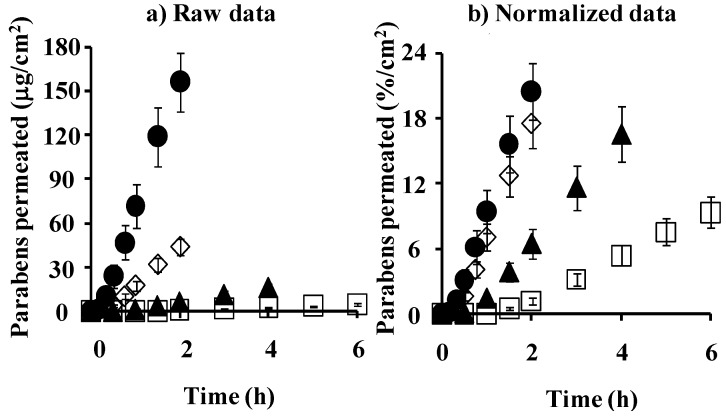
Time course of the cumulative amount of parabens that permeated hairless rat stripped skin. (**a**) raw data; (**b**) normalized data. ●: 10 mM MP; ◊: 3 mM EP; ▲: 1 mM PP; □: 0.5 mM BP. Each data point represents the mean ± S.E. (*n* = 3).

First, the stripped skin permeability of parabens was measured. Figure 10a,b show raw and normalized permeation profiles through stripped skin. Tape stripping of the stratum corneum increased the skin permeation of parabens, especially hydrophilic parabens such as MP.

**Figure 11:**
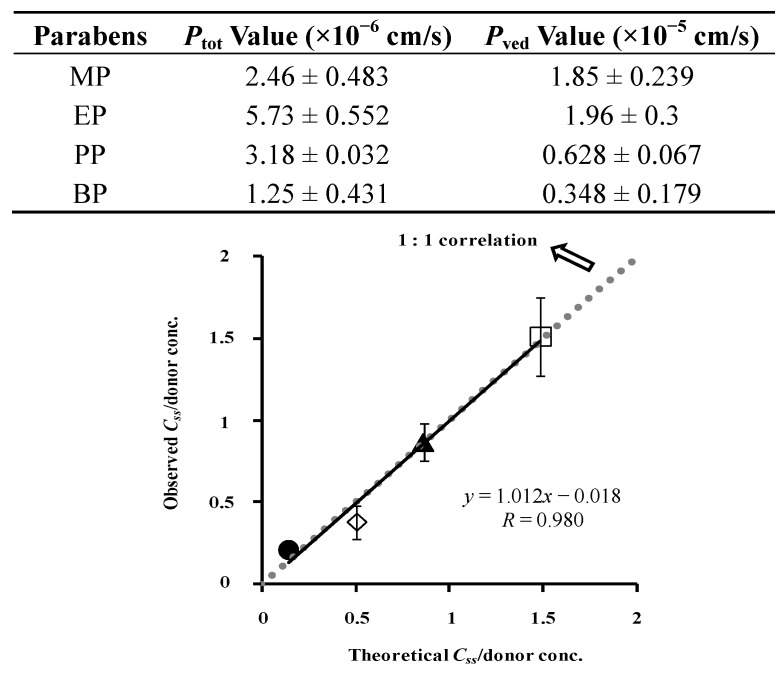
Relationship between theoretical and observed *C*_ss_ in hairless rat skin. Normalized data were used (see Figure 7). Two-layered diffusion membrane model was used to obtain theoretical steady-state skin concentration of parabens. Symbols: as in Figure 3. The observed data represent the mean ± S.D. (*n* = 3–8). Almost 1:1 correlation was found. *P*_tot_ and *P*_ved_ values were listed below.

Figure 11 shows the relation between the observed and calculated skin concentrations. The figure also summarizes the obtained permeation parameters of parabens in the legend. The theoretical values are close to the observed values, although little difference was found, which may be dependent on the process of washing the skin surface. The two-layered model predicts the skin concentration of parabens from skin permeation experiments much better than the one-layered model of hairless rat skin. Animal skin has appendages such as hair follicles and sweat ducts as additional permeation pathways to the primary permeation pathway, the stratum corneum [21]. The hydrophilic pathway as well as the lipophillic pathway play a role in the overall skin permeation of several compounds. The contribution of appendages and the hydrophilic pathway must be taken into account to better predict the skin concentration of materials from skin permeation profiles.

**Figure 12:**
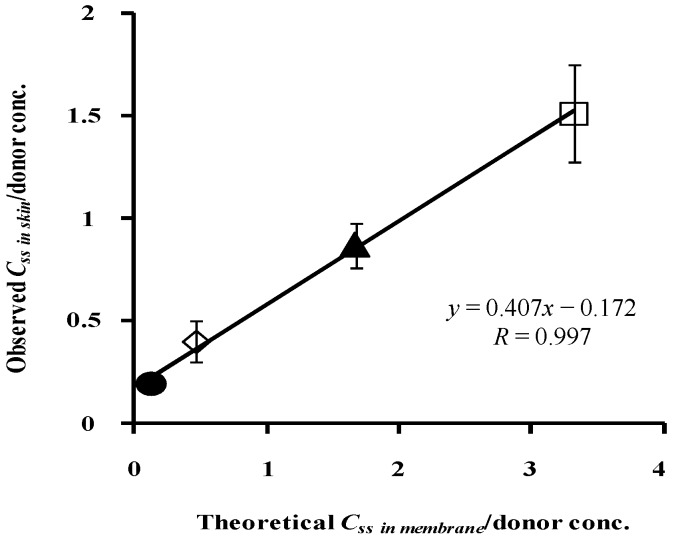
Relationship between theoretical *C*_ss_ in silicone membrane/donor concentrationand observed *C*_ss_ in rat skin/donor concentration. ●: 10 mM MP; ◊: 3 mM EP; ▲: 1 mM PP; □: 0.5 mM BP. Each data point represents the mean ± S.D. (*n* = 3–8).

Figure 12 shows the relationship between the theoretical concentration of parabens in the silicone membrane and the normalized observed concentration of parabens in hairless rat skin. The very high correlation coefficient, 0.997, between them suggests the high predictability of the skin concentration of parabens using silicone membrane permeation experiments.

We supposed a homogenous one-layered model for the silicone membrane and two-layered model consisted of stratum corneum and the following layer for the rat skin. Although these membrane models were different, their concentration-distance profiles and theoretical concentrations of drug or cosmetic ingredient in both the membranes can be expressed only by physical diffusion model. Thus, membrane concentration in the two-layered diffusion model can be easily replaced by that in the one-layered model using a mathematical approach. This is a reason why diffusion profile through silicone membrane is useful to predict the skin concentration of drugs or cosmetic ingredients.

In the near future, we plan to use broad compounds other than parabens, which is a simple series of compounds. We also plan to use several topical formulations, such as creams, ointments and patches. A silicone membrane permeation study using broad compounds from several formulations will produce a monogram of how to estimate the skin concentration of materials. All topical drugs have different target sites in skin tissues. Distribution of the skin concentration must be clarified from the shallow to deep layer in the near future. Since this is an alternative method to animal studies, it can be easily used by pharmaceutical and cosmetic companies to estimate the skin concentration after applying topical drug formulations and cosmetics.

## 5. Conclusions

The drug concentration in the silicone membrane and animal skin can be easily predicted using diffusion equations and membrane permeation data. This method can be applied to the design of cosmetic and topical pharmaceutical formulations. A silicone membrane can be used as an alternative membrane to animal skin.
